# Emerging reservoirs of metallo-β-lactamase genes among non-*aeruginosa Pseudomonas* and *Stutzerimonas* species in a Spanish tertiary hospital

**DOI:** 10.3389/fmicb.2026.1810036

**Published:** 2026-05-22

**Authors:** Guillem Puigsech-Boixeda, Albert Moreno-Mingorance, Ester del Barrio-Tofiño, Virginia Rodríguez-Garrido, Alba Mir-Cros, Josep Roca-Grande, Xavier Nuvials, Dolors Rodríguez-Pardo, Agustín Gayubas, Sílvia Aneas, Adaia Albasanz-Puig, Yannick Hoyos-Mallecot, Jesús Trejo-Zahinos, Mayli Lung, Paula Salmerón, M. Teresa Martín-Gómez, Daniel Romero-Herrero, Belén Viñado, M. Nieves Larrosa, Juan José González-López

**Affiliations:** 1 Vall d'Hebron Institut de Recerca, Vall d'Hebron Barcelona Hospital Campus, Barcelona, Spain; 2 Genetics and Microbiology Department, Universitat Autònoma de Barcelona, Bellaterra, Spain; 3 CIBER de Enfermedades Infecciosas (CIBERINFEC), Instituto de Salud Carlos III, Madrid, Spain; 4 Clinical Microbiology Department, Hospital Universitari Vall d'Hebron, Barcelona, Spain; 5 Critical Care Department, Hospital Universitari Vall d'Hebron, Barcelona, Spain; 6 Infectious Diseases Department, Hospital Universitari Vall d'Hebron, Barcelona, Spain; 7 Preventive Medicine Department, Hospital Universitari Vall d'Hebron, Barcelona, Spain; 8 Medicine Department, Universitat Autònoma de Barcelona, Bellaterra, Spain

**Keywords:** antimicrobial resistance, carbapenemase, genomic epidemiology, metallo-β-lactamase, mobile genetic elements, *Pseudomonas*, *Stutzerimonas*, whole-genome sequencing

## Abstract

**Introduction:**

*Pseudomonas* is one of the largest genera among Gram-negative bacteria, currently comprising nearly 400 valid species. Among them, non-*aeruginosa Pseudomonas* species, together with *Stutzerimonas*—a genus recently separated from *Pseudomonas*—are becoming important reservoirs of antimicrobial resistance genes. However, accurate species-level identification of these organisms remains challenging when relying on the methods routinely implemented in most clinical microbiology laboratories, such as MALDI-TOF mass spectrometry. Therefore, this study aimed to identify and characterize metallo-β-lactamase-producing non-*aeruginosa Pseudomonas* spp. and *Stutzerimonas* spp. isolates obtained in a Spanish tertiary hospital, focusing on their species-level identification, dissemination and role as reservoirs of carbapenemase-encoding genes.

**Methods:**

Whole-genome sequencing (WGS) and antimicrobial susceptibility testing (AST) were used to identify and characterize a total of 22 carbapenemase-producing non-*aeruginosa Pseudomonas* and *Stutzerimonas* isolates collected from patient and environmental samples from March 2022 to July 2024 in a Spanish tertiary hospital.

**Results:**

WGS analysis identified eight species: *Pseudomonas alloputida* (*n* = 7), *Pseudomonas mosselii* (*n* = 5), *Pseudomonas kurunegalensis* (*n* = 4), *Pseudomonas asiatica* (*n* = 2), *Pseudomonas oleovorans* (*n* = 1), *Pseudomonas soli* (*n* = 1), *Stutzerimonas chloritidismutans* (*n* = 1) and *Stutzerimonas stutzeri* (*n* = 1). Multilocus sequence typing (MLST) classified isolates of the *P. putida* group into nine STs, revealing three predominant clones: *P. alloputida* ST69, *P. mosselii* ST115 and *P. kurunegalensis* ST114. AST results showed resistance rates >55% to all tested β-lactams, aminoglycosides and fluoroquinolones, except for cefiderocol, amikacin and colistin (0–5%). The following carbapenemase combinations were detected: *bla*_VIM−1_ (*n* = 16), *bla*_VIM−1_+*bla*_VIM−2_ (*n* = 4), *bla*_VIM−1_+*bla*_NDM−1_ (*n* = 1) and *bla*_VIM−11_ (*n* = 1). Seven distinct *bla*_VIM_-harboring plasmids were detected in 16 isolates, whereas the remaining six carried chromosomally-integrated *bla*_VIM_. Eight different *bla*_VIM_-harboring class I integrons were detected, two of which also occurred in *P. aeruginosa* clinical isolates obtained in the same hospital.

**Conclusion:**

This study highlights the epidemiological relevance of non-*aeruginosa Pseudomonas* and *Stutzerimonas* species as reservoirs of metallo-β-lactamase genes encoded in mobile genetic elements. These findings reinforce the need for systematic WGS-based surveillance of both clinical and environmental isolates, enabling the detection of hidden transmission networks and interspecies dissemination of antimicrobial resistance determinants.

## 1 Introduction

*Pseudomonas* is one of the largest genus among Gram-negative bacteria and hosts an extensive genetic diversity, currently comprising nearly 400 valid species (Göker et al., 2025). These species have been classified into 16 groups, with the *Pseudomonas putida* and *Pseudomonas fluorescens* groups being the largest, including 51 and 48 valid species respectively (Girard et al., 2021; Urbanowicz et al., 2022). Nonetheless, the increasing use of whole-genome sequencing (WGS) is enabling more precise species classifications, resulting in significant changes in taxonomy. One example is *Pseudomonas stutzeri*, a species that has recently been reclassified into a whole new genus designated *Stutzerimonas* (Lalucat et al., 2022; Oren and Garrity, 2022).

In addition to *Pseudomonas aeruginosa*, several species within the genus *Pseudomonas*, and more recently, members of the *Stutzerimonas* genus, have been reported as causative agents of opportunistic infections or colonization in humans, with the majority belonging to the *P. putida* group. Species of this group are globally distributed and predominantly associated with diverse environmental niches including soil, freshwater, and industrial environments (Lalucat et al., 2006; Wu et al., 2011). Despite their classification as low-incidence opportunistic pathogens, they represent a significant reservoir of antimicrobial resistance genes, including metallo-β-lactamases (MBLs). These enzymes efficiently hydrolyze most β-lactam antibiotics and are not inhibited by currently available β-lactamase inhibitors. Within MBLs, VIM and IMP types are the variants most frequently found in *Pseudomonas* species (Juan et al., 2010; Bashar et al., 2017; Brovedan et al., 2021). In particular, *bla*_VIM_ is typically found encoded in class I integrons embedded in transposons that harbor additional resistance genes, generally related to aminoglycoside resistance (Liu et al., 2025).

Among the species included in the *P. putida* group, a small subset has repeatedly been associated with nosocomial infections or colonization. These species include *P. putida* (Tan et al., 2019), *P. mosselii* (Leneveu-Jenvrin et al., 2013), *P. alloputida* (Urbanowicz et al., 2022), *P. monteilii* (Tohya et al., 2021), *P. asiatica* (Tohya et al., 2019), *P. kurunegalensis* (Urbanowicz et al., 2022) and *P. juntendi* (Urbanowicz et al., 2022). Nevertheless, for most of these species, the broader clonal population structures and potentially epidemic genotypes remain largely unexplored. A limited number of recent studies have employed WGS to accurately identify clinical carbapenemase-producing isolates belonging to this group, with reports from Poland (Urbanowicz et al., 2022), Brazil (Alberto-Lei et al., 2022), Argentina (Brovedan et al., 2021), Japan (Tohya et al., 2019), and China (Lin et al., 2024). In Spain, the clinical relevance of these species is considered minimal due to their overall low incidence, and few studies have explored their importance as a reservoir of antimicrobial resistance genes. These studies have specifically been focused on *P. monteilii* (Ocampo-Sosa et al., 2015), *P. putida* (Juan et al., 2010), and *P. kurunegalensis* (Badenas-Alzugaray et al., 2025).

Regarding *Stutzerimonas* species, a few studies have accurately identified clinical carbapenemase-producing isolates using WGS, including a recent report from China (Shi et al., 2024). In this context, the inclusion of *Stutzerimonas* species, which are phylogenetically close to *Pseudomonas* and share similar ecological and clinical niches, would further expand our understanding of highly related non-*aeruginosa* pseudomonads as emerging carriers of carbapenemase genes.

Therefore, the main objective of this study was the identification and genomic characterization of MBL-producing non-*aeruginosa Pseudomonas* spp. (MBL-NAP) and *Stutzerimonas* spp. isolates (MBL-STZ) identified in the Hospital Universitari Vall d'Hebron (Barcelona), in order to determine their dissemination and significance as reservoirs of carbapenemase-encoding genes.

## 2 Materials and methods

### 2.1 Study population and bacterial isolates

From March 2022 to July 2024, all the MBL-NAP and MBL-STZ isolates obtained from patient samples and environmental studies in the Hospital Universitari Vall d'Hebron (Barcelona) were included in this study. In addition, 20 *bla*_VIM_-harboring *P. aeruginosa* isolates obtained at our hospital between 2022 and 2024, were included for a comparative analysis of *bla*_VIM_ genetic environments; these isolates differed by more than 9 core genome multilocus sequence typing (cgMLST) alleles to exclude clonality.

### 2.2 Bacterial culture, identification, and antibiotic susceptibility testing

*Pseudomonas* spp. and *Stutzerimonas* spp. isolates were identified by mass spectrometry (MALDI-TOF, Vitek-MS, *in vitro* diagnostic knowledge database version 3.3.0, bioMérieux, Spain) after growth in standard culture media (i.e. Columbia agar + 5% sheep blood (bioMérieux), CHROMID^®^ CPS^®^ Elite Agar (bioMérieux), MacConkey Agar + Cefotaxime (Thermo Scientific™), CHROMID^®^ CARBA SMART Agar (bioMérieux), among others). All cultures were incubated at 37 °C for 24 h. Susceptibility to piperacillin/tazobactam (range: 4/4 mg/L−64/4 mg/L), ceftazidime (range: 1 mg/L−32 mg/L), cefepime (range: 1 mg/L−32 mg/L), ceftolozane/tazobactam (range: 0.5/4 mg/L−8/4 mg/L), aztreonam (range: 0.5 mg/L−32 mg/L), aztreonam/avibactam (range: 0.03/4 mg/L−64/4 mg/L), imipenem (range: 1 mg/L−64 mg/L), meropenem (range: 0.5 mg/L−32 mg/L), amikacin (range: 2 mg/L−32 mg/L), gentamicin (range: 1 mg/L−16 mg/L), tobramycin (range: 1 mg/L−16 mg/L), ciprofloxacin (range: 0.12 mg/L−2 mg/L), levofloxacin (range: 0.5 mg/L−4 mg/L) and colistin (range: 0.5 mg/L−8 mg/L) were assessed by microdilution using Sensititre^TM^ YEUX2NF and YEUAZAXF panels (Thermo Fisher, Waltham, MA, USA), according to the manufacturer's recommendations. Cefiderocol susceptibility testing was performed by disk diffusion on Mueller-Hinton agar with cefiderocol 30 μg discs (Liofilchem^®^) according to EUCAST recommendations. The results were interpreted according to EUCAST v15.0 (European Committee on Antimicrobial Susceptibility Testing, 2025) criteria for *Pseudomonas* spp. Due to the lack of specific breakpoints for ceftolozane/tazobactam and cefiderocol in *Pseudomonas* spp. other than *P. aeruginosa*, EUCAST breakpoints for *P. aeruginosa* were applied. Additionally, CLSI (Clinical and Laboratory Standards Institute, 2025) recommendations under the category “Other non-Enterobacterales” were used for gentamicin interpretation. In the case of aztreonam/avibactam, interpretation was based on *Pseudomonas* spp. EUCAST breakpoints for aztreonam. Carbapenemase production was confirmed by a lateral flow immunoassay (NG-Test Carba 5 assay; NG-Biotech, ZI Courbouton, France).

### 2.3 Whole-genome sequencing and bioinformatic analysis

Short-read and long-read sequencing were performed in all the MBL-NAP and MBL-STZ isolates. DNA was extracted using the DNeasy UltraClean Microbial Kit (QIAGEN, Hilden, Germany). For short-read sequencing, libraries were prepared using the Illumina DNA Library Prep Kit and sequenced using the MiSeq device (Illumina, USA), according to the manufacturer's instructions. Trimmomatic v0.39 (Bolger et al., 2014) and Unicycler v0.4.8 (Wick et al., 2017) were used for raw-read trimming and *de novo* genome assembly. Long-read bacterial genome sequencing was performed using Oxford Nanopore Technology, with Filtlong v0.2.1 (https://github.com/rrwick/filtlong), Flye v2.9.1 (Kolmogorov et al., 2019) and Medaka v1.8.0 (https://github.com/nanoporetech/medaka) for raw read trimming, *de novo* genome assembly and assembly polishing, respectively.

Genome-based species identification was performed using the Ribosomal MLST analysis tool available at PubMLST (Jolley et al., 2018) and JSpeciesWS (Richter et al., 2016), which conducted ANIm pairwise comparisons. MLST was performed in the species belonging to the *P. putida* group by submitting genome assemblies to PubMLST. For the remaining species, MLST was not performed as no schemes are currently available.

Acquired genetic determinants of antimicrobial resistance were identified with Resistance Gene Identifier v6.0.3 (https://card.mcmaster.ca/analyze/rgi). Annotation of the sequenced genomes was performed using Bakta v1.9.4 (Schwengers et al., 2021) and ISfinder (Siguier et al., 2006). Plasmid structural analysis and graphical representation were carried out using Geneious Prime v2024.0.7 (https://www.geneious.com). Integron nomenclature followed the *In* numbering system for class I integrons proposed in Integrall (Moura et al., 2009). Plasmid incompatibility groups were assigned based on homology of replication, partition, mobilization and conjugation modules to described plasmid families using BLASTn (https://blast.ncbi.nlm.nih.gov/Blast.cgi).

Single-nucleotide polymorphism (SNP) analysis was carried out with Snippy v4.3.6 (https://github.com/tseemann/snippy), using as genome references the long-read annotated genomes from the study isolates P03 for *P. alloputida*, P22 for *P. mosselii* and P07 for *P. kurunegalensis* (Supplementary Table 1), corresponding to isolates of the same sequence type (ST) as the isolates included in each respective phylogeny, allowing precise visualization of phylogenetic relationships. Recombination events were removed with Gubbins v2.3.4 (Croucher et al., 2014). Finally, a maximum-likelihood phylogeny was constructed after 1,000 bootstrap replicates with IQ-Tree v1.6.10 (Nguyen et al., 2014) and tree annotation with relevant metadata was performed with interactive tree of life (iTOL) (Letunic and Bork, 2016).

### 2.4 Comparative analysis of *bla*_*VIM*_ genetic environments with VIM-producing *P. aeruginosa*

In order to investigate the potential presence of similar *bla*_VIM_-harboring plasmids or integron structures in *P. aeruginosa* isolates circulating in our local epidemiology, the genetic environments of *bla*_VIM_ detected in the MBL-NAP and MBL-STZ isolates were compared with those of 20 VIM-producing *P. aeruginosa* isolates obtained at our hospital during the study period. Data from the *P. aeruginosa* isolates, including sample source, year of isolation, ST, AST results and carbapenemases detected, can be found in Supplementary Table 2.

Comparative analyses were performed with Snippy v4.3.6 and BLASTn, using the sequence of the *bla*_VIM_-harboring plasmids and integrons detected in the MBL-NAP and MBL-STZ isolates as reference.

### 2.5 Comparative analysis of *bla*_*VIM*_-harboring plasmids with previous studies

In order to identify previously described *bla*_VIM_-harboring plasmids similar to the plasmids identified in our study, alignment was performed using BLASTn in core nucleotide and RefSeq Genome databases, filtering for sequences sharing at least 90% coverage and 90% nucleotide identity.

## 3 Results

### 3.1 Bacterial isolates and patient clinical background

A total of 20 MBL-NAP and 2 MBL-STZ isolates obtained from March 2022 to July 2024 were included in the study. Of these, 21 were obtained from 20 patients attended in the Hospital Universitari Vall d'Hebron (Barcelona) and one isolate was from an environmental study of a sink drain in the intensive care unit (ICU).

All the isolates were initially identified using MALDI-TOF as: *P. putida* (*n* = 13), *P. mosselii* (*n* = 6), *S. stutzeri* (*n* = 2) and *P. oleovorans*. (*n* = 1). After WGS analysis, the 13 isolates initially identified by MALDI-TOF as *P. putida* were mostly reclassified as *Pseudomonas alloputida* (*n* = 7/13), followed by *Pseudomonas kurunegalensis* (*n* = 4/13) and *Pseudomonas asiatica* (*n* = 2/13). Among the six isolates initially identified as *P. mosselii*, genome analysis confirmed that all but one were *P. mosselii* (*n* = 5/6), while the remaining isolate was identified as *Pseudomonas soli* (*n* = 1/6). In the case of the two isolates identified as *S. stutzeri*, one was confirmed to be a *Stutzerimonas stutzeri* and the other a *Stutzerimonas chloritidismutans*. Finally, the *P. oleovorans* isolate was consistently identified as such by both methods.

All *P. alloputida, P. kurunegalensis* and *P. oleovorans* isolates were obtained from urine samples. Among the five *P. mosselii* isolates, two were obtained from urine samples, while the remaining three were obtained from different sources, including a tracheal aspirate, a rectal swab, and an environmental sample from an ICU sink drain. In the case of the two *P. asiatica* isolates, one was recovered from an articular biopsy and the other from a rectal swab. Finally, *P. soli, S. stutzeri* and *S. chloritidismutans* isolates were obtained from a catheter, a blood clot from a surgical wound and pleural fluid, respectively. Details of the sample origin of each isolate are provided in Supplementary Table 1.

All patient-derived isolates belonged to different individuals except one *P. mosselii* and one *P. asiatica*, which were obtained from a same patient (isolates P20 and P21, Supplementary Table 1). These isolates were obtained from patients with a broad age range (1–83 years), with a median age of 55 years. Most patients had underlying conditions linked to immunodeficiency or immunosuppression, with the majority being transplant recipients (*n* = 7/20) or presenting neoplasia or leukemia (*n* = 7/20). The remaining patients had diverse conditions but all related to prolonged hospitalization, including disorders such as tracheobronchitis, and cerebral aneurysm, among others (Supplementary Table 1).

### 3.2 Antimicrobial susceptibility

AST results showed that all the MBL-NAP and MBL-STZ isolates were resistant to all the β-lactams tested except for aztreonam, aztreonam/avibactam, and cefiderocol, which showed a resistance rate of 59%, 59%, and 0%, respectively. Notably, aztreonam/avibactam exhibited the same activity as aztreonam alone, with no changes in minimum inhibitory concentration (MIC) values observed in the presence or absence of avibactam. The resistance rates per species to β-lactams, aminoglycosides, fluoroquinolones and colistin are shown in Table 1. The MIC values for each isolate and tested antimicrobial are shown in Supplementary Table 1.

**Table 1:** Number of resistant isolates and antimicrobial resistance rates per antimicrobial agent among MBL-NAP and MBL-STZ isolates.

Antimicrobial agent	*P. alloputida* (*n =* 7)	*P. mosselii* (*n =* 5)	*P. kurunegalensis* (*n =* 4)	*P. asiatica* (*n =* 2)	*S. chloritidismutans* (*n =* 1)	*S. stutzeri* (*n =* 1)	*P. soli* (*n =* 1)	*P. oleovorans* (*n =* 1)	Total (*n =* 22) *n* (%)
Piperacillin/tazobactam	7	5	4	2	1	1	1	1	22 (100)
Ceftolozane/tazobactam	7	5	4	2	1	1	1	1	22 (100)
Ceftazidime	7	5	4	2	1	1	1	1	22 (100)
Cefepime	7	5	4	2	1	1	1	1	22 (100)
Cefiderocol	0	0	0	0	0	0	0	0	0
Aztreonam	7	0	2	2	1	1	0	0	13 (59)
Aztreonam/avibactam	7	0	2	2	1	1	0	0	13 (59)
Imipenem	7	5	4	2	1	1	1	1	22 (100)
Meropenem	7	5	4	2	1	1	1	1	22 (100)
Amikacin	0	1	0	0	0	0	0	0	1 (5)
Gentamicin	4	1	2	1	1	1	1	1	12 (55)
Tobramycin	4	1	4	2	1	1	1	1	15 (68)
Ciprofloxacin	7	2	4	2	0	1	1	0	17 (77)
Levofloxacin	7	2	4	2	0	1	1	0	17 (77)
Colistin	0	0	0	0	0	0	0	0	0

Results were interpreted according to EUCAST 2025 clinical breakpoints for MIC interpretation in *Pseudomonas* spp., except for ceftolozane/tazobactam and cefiderocol, for which EUCAST 2025 *P. aeruginosa* breakpoints were used, and gentamicin, which was interpreted according to CLSI breakpoints for other non-Enterobacterales.

Antimicrobial resistance gene content analysis revealed that all the isolates harbored *bla*_VIM−1_ except for one *P. asiatica*, which carried *bla*_VIM−11_. Specifically, 72% of the isolates harbored *bla*_VIM−1_ (16/22), 18% *bla*_VIM−1_ + *bla*_VIM−2_ (4/22), 5% *bla*_VIM−1_ + *bla*_NDM−1_ (1/22) and 5% *bla*_VIM−11_ (1/22). Additionally, two *P. kurunegalensis* isolates encoded *bla*_OXA − 2_ and one *P. alloputida* harbored *bla*_TEM−110_. Interestingly, all the *P. kurunegalensis* isolates simultaneously encoded *bla*_VIM−1_ and *bla*_VIM−2_, and one *P*.*mosseliibla*_VIM−1_ and *bla*_NDM−1_. All gentamicin and/or tobramycin resistant isolates encoded *aac(6*′*)-Ib4-*like (99% amino acid identity to *aac(6*′*)-Ib4*), and the amikacin resistant *P. mosselii* harbored *rmtC*. Regarding fluoroquinolone-resistant isolates, 94% (16/17) presented the T83I substitution in the quinolone resistance-determining region (QRDR) of *gyrA*, and the remaining isolate harbored *qnrVC1* (Supplementary Table 1).

### 3.3 Genomic epidemiology and phylogenetic analysis

**Figure 1:**
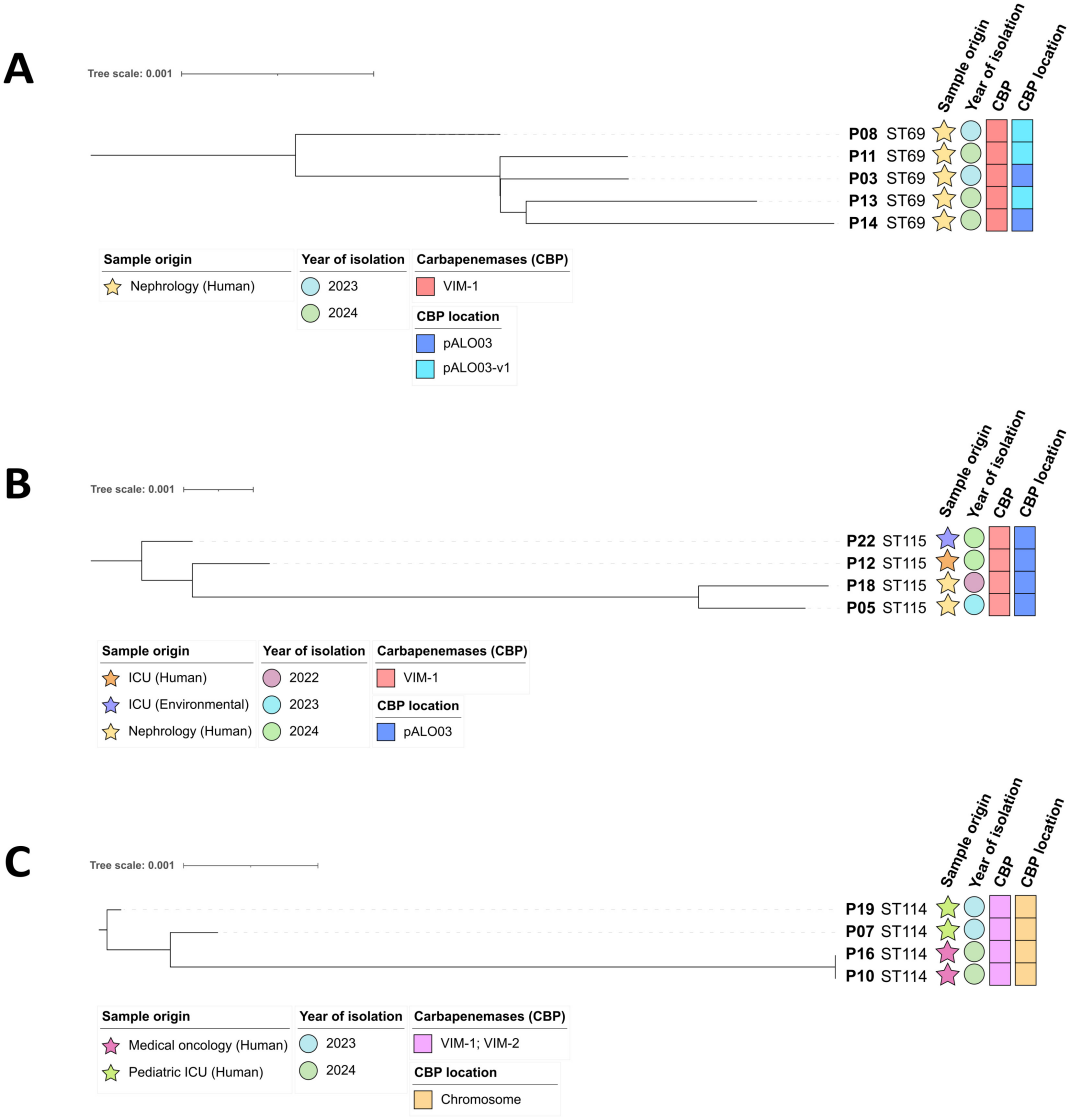
Maximum-likelihood tree and phylogenomic analysis of isolates belonging to *P. alloputida* ST69 **(A)**, *P. mosselii* ST115 **(B)**, and *P. kurunegalensis* ST114 **(C)**. The reference strain for its construction was P03 for *P. alloputida*, P22 for *P. mosselii* and P07 for *P. kurunegalensis*. The characteristics of the isolates, including ST, year of isolation, sample origin, carbapenemases and its genomic location are shown to the right of the phylogeny.

MLST analysis was performed in isolates belonging to species of the *P. putida* group, including *P. alloputida* (*n* = 7), *P. mosselii* (*n* = 5), *P. kurunegalensis* (*n* = 4), *P. asiatica* (*n* = 2) and *P. soli* (*n* = 1). Subsequently, a maximum-likelihood phylogeny was constructed for isolates belonging to the same species and ST (Figure 1).

Regarding *P. alloputida*, five isolates encoding *bla*_VIM−1_ belonged to ST69 (P03, P08, P11, P13, and P14), while the remaining two *bla*_VIM−1_-harboring isolates belonged to ST153 (P04) and to the novel ST309 (P09), respectively. The median genetic distance observed among the isolates belonging to ST69 was 18 SNPs (SNP range: 2–25) and these isolates were obtained from patients admitted to the Nephrology Unit, with no temporal overlap between them (Figure 1A).

In the case of *P. mosselii*, four isolates encoding *bla*_VIM−1_ were assigned to ST115 (P05, P12, P18, and P22), while the remaining isolate, which encoded *bla*_VIM−1_ and *bla*_NDM−1_, belonged to ST36 (P20). The median genetic distance observed among isolates belonging to ST115 was 147 SNPs (SNP range: 24–265) (Figure 1B). Among these, isolates P05 and P18 differed by 24 SNPs, and were obtained from patients admitted to the Nephrology Unit with no temporal overlap between them. In contrast, isolates P12 and P22 differed by 30 SNPs and were both obtained from the ICU. Interestingly, P22 originated from an environmental sample collected from an ICU sink drain, while P12 was recovered from a patient admitted to the ICU during the same month the environmental sample was obtained.

Regarding *P. kurunegalensis*, all the isolates encoded *bla*_VIM−1_ and *bla*_VIM−2_, and belonged to ST114 (P07, P10, P16, and P19). The median genetic distance observed among them was 85 SNPs (SNP range: 0–86) (Figure 1C). Notably, isolates P07 and P19 differed by 5 SNPs and were recovered from two patients hospitalized in the pediatric ICU who had temporally overlapped within that unit. Similarly, isolates P10 and P16 showed a genetic distance of 0 SNPs and were obtained from two patients admitted to the Medical Oncology Unit, who had also coincided temporally.

In the case of *P. asiatica*, the *bla*_VIM−11_-encoding isolate P17 belonged to ST97, while the *bla*_VIM−1_-encoding isolate P21 was assigned to the novel ST310. Finally, the *bla*_VIM−1_-encoding *P. soli* isolate P01 was assigned to ST104.

### 3.4 Genetic environment of the MBL-encoding genes

Genomic annotation of the assembled long reads showed that 100% of the isolates carried *bla*_VIM_ genes on class I integrons, with 73% of these (16/22) carrying it on a plasmid and the remaining 27% (6/22) integrated into the chromosome. None of the isolates encoded *bla*_VIM_ simultaneously in both genomic structures. Chromosomally integrated *bla*_VIM_ were detected in all *P. kurunegalensis* isolates, as well as in one *P. alloputida* and one *P. mosselii* (Supplementary Table 1). Regarding the *P. mosselii* isolate encoding *bla*_NDM−1_, it was found integrated in the chromosome.

Plasmid analysis identified seven different plasmids among the 16 isolates harboring plasmid-encoded *bla*_VIM_, called pSOL01, pSTU02, pALO03, pSTU06, pOLE015, pASI017, and pASI021 (Supplementary Figures 1–7). All seven plasmids harbored *bla*_VIM_ within unique class I integrons, with the exception of plasmids pSOL01 and pSTU06, which shared an identical integron In488, featuring the gene cassette array (GCA) 5′CS-*bla*_VIM−1_-*aac(6*′*)-Ib4*-like-*aadA1*-3′CS. In the remaining plasmids, the following *bla*_VIM_-harboring integron structures were identified: novel integron InVH-P03 (GCA: 5′CS-*bla*_VIM−1_-*aac(6*′*)-Ib4*-like-*bla*_VIM−1_-*aadA1*-3′CS) in pALO03, In70 (GCA: 5′CS-*bla*_VIM−1_-*aac(6*′*)-Ib4*-like-*aphA15*-*aadA1*-3′CS) in pSTU02, In334 (GCA: 5′CS-*bla*_VIM−1_-*aac(6*′*)-Ib4*-like-3′CS) in pOLE015, novel integron InVH-P17 (GCA: 5′CS-*aacA9*-like-*bla*_VIM−11_-3′CS) in pASI017, and novel integron InVH-P21 (GCA: 5′CS-*aac(6*′*)-Ib4*-like-*bla*_VIM−1_-*aac(6*′*)-Ib4*-like-*aadA1*-*catB2*-3′CS) in pASI021. Additionally, a pALO03 plasmid variant (pALO03-v1) lacking one *bla*_VIM−1_ gene copy and *aac(6*′*)-Ib4-*like in integron InVH-P03 was detected in *P. alloputida* isolates susceptible to gentamicin and tobramycin.

Each of these plasmids was found in single isolates, with the exception of pALO03, which was detected in two ST69 (2/5) and one ST153 *P. alloputida* isolates, and also in all four ST115 *P. mosselii* isolates, including the one obtained from the environmental ICU sample (Supplementary Table 1). Interestingly, although ST69 *P. alloputida* isolates showed a close genetic distance of 2–25 SNPs, the remaining three ST69 isolates (3/5) harbored *bla*_VIM−1_ within a pALO03-v1 plasmid variant (Figure 1). Regarding the other plasmids identified, pSOL01 was found in *P. soli*, pSTU02 in *S. stutzeri*, pSTU06 in *S. chloritidismutans*, pOLE015 in *P. oleovorans*, and pASI017 and pASI021 in *P. asiatica*. Plasmid pOLE015 belonged to the IncP6 family, whereas the remaining plasmids could not be attributed to any previously described replicon group.

**Figure 2:**
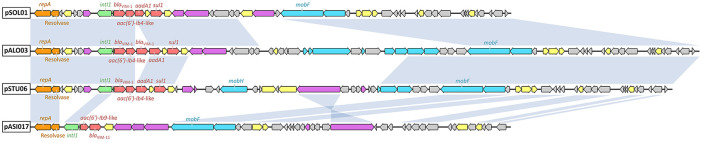
Structural organization of pSOL01, pALO03, pSTU06, and pASI017 plasmids. The genes appear color-coded according to their function as follows: replication (orange), transposition (purple), integration (green), antimicrobial resistance (red), plasmid transference (light blue), other functions (yellow) and hypothetical (gray). Blue shading denotes shared regions of identity.

Among all *bla*_VIM_-encoding plasmids analyzed, pSOL01, pALO03, pSTU06, and pASI017 shared at least 53% coverage and 99.9% identity, harboring identical *mobF* and *repA* genes. However, they showed structural differences primarily due to deletions within the coding regions of plasmid transfer genes, as well as variations in the *bla*_VIM_-harboring integrons (Figure 2).

**Figure 3:**
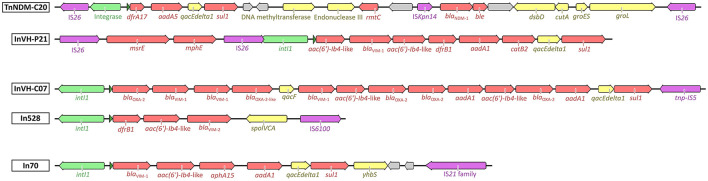
Genetic environment of MBLs integrated into the chromosome in *P. mosselii* isolate P20 (TnNDM-C20 and InVH-P21), *P. kurunegalensis* isolates P07 and P19 (InVH-C07 and In528), *P. kurunegalensis* isolates P10 and P16 (InVH-P21 and In528), and *P. alloputida* isolate P09 (In70). The genes appear color-coded according to their function using the same criteria as in Figure 2.

Regarding chromosomally integrated MBLs, *P. mosselii* isolate P20 harbored *bla*_NDM−1_ within a composite transposon formed by IS*26* transposases that also carried additional resistance genes, including *dfrA17, aadA5, sul1* and *rmtC* (TnNDM-C20, Figure 3). Additionally, this isolate harbored *bla*_VIM−1_ within the InVH-P21-like integron, showing its integrase gene truncated as a result of the insertion of another IS*26* composite transposon harboring *mphE* and *msrE* (Figure 3).

In the case of *P. kurunegalensis*, isolates P07 and P19 encoded *bla*_VIM−1_ in InVH-C07, which, interestingly, featured multiple integration of *bla*_VIM−1_, *bla*_OXA − 2_ and *aac(6*′*)-Ib4*-like gene cassettes (Figure 3), while the remaining isolates P10 and P16 harbored *bla*_VIM−1_ within InVH-P21, also found in plasmid pASI021. Concerning *bla*_VIM−2_, all *P. kurunegalensis* isolates (P07, P10, P16, and P19) carried it within the In528-like structure (GCA: 5′CS-*dfrB1-aac(6*′*)-Ib4-*like-*bla*_VIM−2_-3′CS) as its 3′CS region was disrupted by IS*6100*. Finally, *P. alloputida* isolate P09 harbored *bla*_VIM−1_ within In70 (GCA: 5′CS-*bla*_VIM−1_-*aac(6*′*)-Ib4*-like-*aphA15*-*aadA1*-3′CS) (Figure 3).

Interestingly, plasmid pASI021 was found in *P. asiatica* isolate P21, which was co-isolated with *P. mosselii* P20 from the same patient, and both isolates shared InVH-P21, although located in different genetic contexts. In addition, the In70 integron found in the chromosome of *P. alloputida* P09 was also found in plasmid pSTU02 from *S. stutzeri* (Supplementary Figure 2).

### 3.5 Comparative analysis of *bla*_*VIM*_ genetic environments with VIM-producing *P. aeruginosa*

To assess whether the genetic structures encoding MBLs detected in MBL-NAP and MBL-STZ isolates were also present among *P. aeruginosa* isolates circulating within our hospital during the same period, we compared the seven plasmids identified in this study with 20 genomes of VIM-producing *P. aeruginosa* collected from the same institution. The alignment yielded the following ranges of query coverage for each plasmid: pSOL01 15%−50%, pSTU02 6%−13%, pALO03 9%−50%, pSTU06 11%−50%, pOLE015 12%−81%, pASI017 7%−58% and pASI021 4%−21%. Notably, plasmid pOLE015 exhibited the highest coverage in two *P. aeruginosa* isolates (81% each) (PAE09 and PAE011, Supplementary Table 2). Nevertheless, analysis of the genetic context of *bla*_VIM_ in these isolates revealed that both shared the same plasmid backbone while harboring *bla*_VIM−2_ and *bla*_VIM−11_ instead of *bla*_VIM−1_, both encoded in single integrons.

The structures of the *bla*_VIM_-encoding integron detected in the MBL-NAP and MBL-STZ isolates were then compared with those found in the *P. aeruginosa* genomes. Specifically, alignment of the integron InVH-P21 (Figure 3) showed that three *P. aeruginosa* isolates (PAE01, PAE07, and PAE014, Supplementary Table 2) carried *bla*_VIM−1_ within an InVH-P21 displaying at least 100% coverage and 97% identity. The only structural difference observed was that two of the detected *P. aeruginosa* isolates carried *catB3* instead of *catB2*. Additionally, two distinct *P. aeruginosa* isolates harbored *bla*_VIM−1_ within an In488 integron, which was detected in pSOL01 and pSTU06 (PAE02 and PAE08, Supplementary Table 2).

### 3.6 Comparative analysis of *bla*_*VIM*_-harboring plasmids with previous studies

BLASTn analysis was performed to identify previously described *bla*_VIM_-harboring plasmids to which the plasmids identified in our study shared at least 90% coverage and 90% nucleotide identity, revealing that only pSOL01, pALO03, and pASI017 showed this level of similarity to at least one plasmid from the NCBI database. Specifically, pSOL01 displayed 92% coverage and 99.9% identity to four *bla*_VIM−1_-harboring plasmids: pBR-PH17 (CP066307.1), pPC9 (CP003739.1), pMOS94 (MK671725.1), and pMEN15 (MK671727.1). Plasmids pBR-PH17 and pPC9 were detected in *P. putida* isolates obtained from Pakistan and France, respectively, while plasmids pMOS94 and pMEN15 were described in *P. mosselii* and *Pseudomonas mendocina* isolates obtained from Italy. Plasmid pSOL01 from our study was 34 kb in size and harbored *bla*_VIM−1_ within an In488 integron (Supplementary Figure 1), whereas plasmids pBR-PH17, pPC9, pMOS94, and pMEN15 were 50–80 kb in size and harbored *bla*_VIM−1_ in four different integrons: In860-like, In869, In70 and a non-described class I integron, respectively. Despite differences in size and integron structure, all four plasmids shared the same genomic regions with pSOL01, including genes involved in plasmid replication and mobilization such as *repA, mobF* and *traD*.

In the case of pALO03, it showed 100% coverage and 99.9% identity with pMOS94 and pMEN15. However, pALO03 was a 46 kb plasmid carrying *bla*_VIM−1_ within the novel integron InVH-P03 (Supplementary Figure 3), whereas pMOS94 and pMEN15 were 50–55 kb in size and harbored *bla*_VIM−1_ within an In70 and a non-described class I integron, respectively, as mentioned previously. Despite size and integron differences, both pMOS94 and pMEN15 shared >80% of their backbone structure with pALO03, differing mainly in the *bla*_VIM−1_-harboring integron and in the presence of additional transposable elements, mostly associated with mercury resistance.

Finally, pASI017 showed 97% coverage and 95% identity to the *bla*_VIM − 84_-harboring plasmid pL2757hy (CP146842.1), described in a *P. monteilii* isolate from China. However, pASI017 was 33 kb in size and harbored *bla*_VIM−11_ within the novel InVH-P17 (Supplementary Figure 6), while plasmid pL2757hy was 49 kb and carried *bla*_VIM − 84_ within a non-described class I integron disrupted by *tniQ*.

## 4 Discussion

This study provides a comprehensive analysis of the dissemination of MBL-producing non-*aeruginosa Pseudomonas* spp. and *Stutzerimonas* spp. in a Spanish hospital between 2022 and 2024, as well as the role of these species as reservoirs of carbapenemase-encoding genes within mobile genetic elements.

Overall, six different MBL-producing non-*aeruginosa Pseudomonas* spp. were identified, five belonging to the *P. putida* group (*P. alloputida, P. mosselii, P. kurunegalensis, P. asiatica*, and *P. soli*) and one *P. oleovorans*, together with two *Stutzerimonas* spp. (*S. stutzeri* and *S. chloritidismutans*), which were recovered from 20 patients and one environmental sample. The species identification obtained by WGS differed from those provided by MALDI-TOF, which initially classified all isolates into only four species (*P. putida, P. mosselii, P. oleovorans* and *S. stutzeri*). Similarly, a study conducted in Poland analyzed 59 MBL-producing isolates, all initially identified as *P. putida* by MALDI-TOF; however, after WGS and subsequent genomic analysis, these isolates were reclassified into 12 distinct species within the *P. putida* group (Urbanowicz et al., 2022). Such discrepancies highlight the challenges in accurately identifying these species and, consequently, the difficulty in tracking their dissemination in hospital settings, underscoring the need to further assess the potential for more precise species identification using the MALDI-TOF systems currently implemented in most clinical microbiology laboratories.

The AST results in the MBL-NAP and MBL-STZ isolates revealed resistance rates higher than 55% to all tested antibiotics except for cefiderocol, amikacin and colistin, which showed resistance rates of 0–5%. Interestingly, the resistance rates of aztreonam and aztreonam/avibactam resistance rates were both 59%. For every isolate, the aztreonam MIC was identical to that of aztreonam/avibactam MIC. These results indicated that isolates showing resistance to both agents likely harbored aztreonam resistance mechanisms unaffected by avibactam. To our knowledge, no study specifically addressing non-enzymatic aztreonam resistance mechanisms in non-*aeruginosa Pseudomonas* and *Stutzerimonas* species has been published to date, but it has been previously described that the main intrinsic antimicrobial resistance mechanism in *P. putida* is the TtgABC efflux pump, the expression of which is induced upon binding of antimicrobial agents to the TtgR repressor (Terán et al., 2003). Homologous genes related to well-characterized *P. aeruginosa* efflux pumps, such as *mexB, mexF, mexK* and *mexW*, have also been found in the *P. putida* group (Mallick et al., 2025), and efflux pumps linked to disinfectant tolerance, such as ParXY, have been linked to increased resistance to cephalosporins, fluoroquinolones and aminoglycosides (Puja et al., 2020). In addition, efflux systems encoded within mobile genetic elements, such as TMexCD-ToprJ, have recently been reported in the *P. putida* group (Jian et al., 2024; Wang et al., 2025). Unfortunately, a major limitation is that most studies describing these systems lack precise species identification, thereby complicating the understanding of efflux-mediated antimicrobial resistance in each of the non-*aeruginosa Pseudomonas* species. Therefore, the aztreonam and aztreonam/avibactam resistance rates observed in isolates from our study could be explained by the expression and/or overexpression of efflux pumps, but more studies are needed to further elucidate the intrinsic antimicrobial resistance mechanisms present in these species.

All the MBL-NAP and MBL-STZ isolates in our study harbored *bla*_VIM−1_ except one *P. asiatica*, which carried *bla*_VIM−11_, in contrast to most reports in which *bla*_VIM−2_ was the main carbapenemase detected among these species (Ocampo-Sosa et al., 2015; Brovedan et al., 2021; Urbanowicz et al., 2022). Differences in MBL prevalence are consistent with data from our local epidemiology, as a recent surveillance study performed in eight Spanish hospitals identified 33 carbapenemase-producing *Pseudomonas* spp. and seven of them belonged to the *P. putida* group, five of which carried *bla*_VIM−1_ (Hernández-García et al., 2024). Additionally, a few studies performed in other European countries have reported *bla*_VIM−1_ as the main detected carbapenemase within the *P. putida* group. For instance, a study from Germany reported the presence of *bla*_VIM−1_ in 41 *P. putida* strains, identifying isolates harboring either *bla*_VIM−1_, or both *bla*_VIM−1_ and *bla*_VIM−2_ (Peter et al., 2017). In addition, a recent study conducted in Italy described a *bla*_VIM−1_-harboring *P. putida* isolate in an oncohaematological patient (Cortazzo et al., 2023). In the case of *Stutzerimonas* spp., only a few studies including carbapenemase-producing isolates have been published, describing cases involving *bla*_VIM−2_ (Bashar et al., 2017) or *bla*_IMP−1_ (Yan et al., 2001). To our knowledge, no cases involving *bla*_VIM−1_ have been published to date for these species. The same applies to *P. oleovorans*, for which only one study describing the presence of *bla*_VIM−2_ has been found (Faccone et al., 2014). In the case of *P. asiatica*, no other reports have described isolates harboring *bla*_VIM−11_, as this variant has been reported mainly in Enterobacterales (Alizadeh et al., 2020) and *P. aeruginosa* (Elena et al., 2024). Interestingly, *P. asiatica* and related non-*aeruginosa Pseudomonas* species are more commonly associated with *bla*_VIM−2_. Given that VIM-11 differs from VIM-2 by a single amino acid substitution, it could be hypothesized that this variant may have emerged through local microevolution from a VIM-2-like ancestor. However, further studies are needed to better understand the origin and dissemination of this enzyme in non-*aeruginosa Pseudomonas* species. Therefore, even though *bla*_VIM−2_ is considered the most prevalent MBL in *P. putida* group species, recent studies increasingly report high rates of *bla*_VIM−1_ and the detection of other variants. In this scenario, different hypotheses could be made on the changing trend of *bla*_VIM_ variants detected in these species, including the unnoticed expansion in clinical settings of specific non-*aeruginosa Pseudomonas* and *Stutzerimonas* clones or by the acquisition of mobile genetic elements integrated into plasmids adapted to these species, facilitating their persistence and dissemination. This underscores the importance of comprehensive WGS-based species identification and genomic characterization of mobile genetic elements in non-*aeruginosa Pseudomonas* and *Stutzerimonas*, providing insights into changes in their epidemiology.

A total of eight different *bla*_VIM_-harboring class I integrons and seven distinct *bla*_VIM_-harboring plasmids were identified among the 22 MBL-NAP and MBL-STZ isolates, with 16 isolates carrying *bla*_VIM_ on a plasmid and the remaining six integrated into the chromosome. BLASTn analysis of the *bla*_VIM_-harboring plasmids revealed that plasmids pSOL01, pALO03, pSTU06, and pASI017 in our study shared identical *repA* and *mobF* genes with the *bla*_VIM−1_-harboring plasmid pMOS94, described in a *P. mosselii* previously identified in Italy. In addition, the Italian study reported that plasmid pMOS94 belonged to a novel plasmid lineage, showing the closest relatedness to the IncW replicon family, which is responsible for the spreading of antibiotic resistance determinants among enterobacteria. Interestingly, this study searched for plasmids in which the *repA* gene showed 100% identity to pMOS94, finding seven MBL-encoding plasmids belonging to this new lineage which were uniquely described within *Pseudomonas* species (Di Pilato et al., 2019). In contrast, plasmids pSTU02, pOLE015, and pASI021 harbored unique genes related to replication, partition, mobilization and conjugation. Among them, only plasmid pOLE015 could be assigned to a described incompatibility group, belonging to the IncP6 family. Although IncP6 plasmids are naturally isolated from *P. aeruginosa* (Boronin, 1992), their presence has also been documented in several Enterobacterales species (Yao et al., 2017). Even so, given the limited number of studies describing plasmid incompatibility groups typically found in non-*aeruginosa Pseudomonas* and *Stutzerimonas* species, it is crucial to continue monitoring plasmid circulation in these species to assess its specific host range, thereby improving our understanding of their role as reservoirs of transmissible antimicrobial resistance determinants.

Interspecies transmission of antimicrobial resistance genes between the *P. putida* group and *P. aeruginosa* is also underexplored. Our results derived from the genomic comparison of *bla*_VIM_-harboring plasmids and integrons with previously sequenced VIM-producing *P. aeruginosa* isolates from our hospital demonstrated that plasmids would not play a significant role in the transmission of *bla*_VIM_ from non-*aeruginosa Pseudomonas* spp. and *Stutzerimonas* spp. to *P. aeruginosa* or vice versa. This observation is further supported by the absence of significant alignment between *bla*_VIM_-harboring plasmids identified in the MBL-NAP and MBL-STZ isolates and previously described *bla*_VIM_-harboring plasmids in *P. aeruginosa*. However, the finding that 25% (5/20) of the VIM-producing *P. aeruginosa* isolates included in this study contained integron structures sharing 100% coverage and at least 97% identity with integrons InVH-P21 or In488 suggests that interspecies *bla*_VIM−1_ transmission to *P. aeruginosa* or vice versa may have occurred due to transposable elements. Previous studies have demonstrated the successful transmission of carbapenemases between species belonging to the *P. putida* group and *P. aeruginosa*. A study performed in Spain studied 11 *P. aeruginosa* and 8 *P. putida* clinical isolates obtained from a single hospital, and found that both species possessed an identical transposon containing the same *bla*_VIM−2_ integron (Juan et al., 2010). In addition, another study from Argentina studied 13 *bla*_VIM−2_-harboring isolates belonging to the *P. putida* group and found three different *bla*_VIM−2_-harboring integrons, including In41, In899, and In528 embedded in three different transposons. One of the integrons was found in a conjugative plasmid detected in *P. asiatica* and also in a *P. aeruginosa* clinical isolate (Brovedan et al., 2021). However, another study from Germany that compared 59 *bla*_VIM_-harboring isolates belonging to the *P. putida* group with 17 *bla*_VIM_-harboring *P. aeruginosa* isolates stated that the involvement of the *P. putida* group in the current major issue of multidrug resistance in *P. aeruginosa* may not be significant, as no *bla*_VIM_-harboring mobile genetic elements were shared between the *P. putida* group and *P. aeruginosa* isolates (Peter et al., 2017). Therefore, although different roles have been proposed for non-*aeruginosa Pseudomonas* and *Stutzerimonas* species in the transmission of MBLs to *P. aeruginosa* or vice versa, our study is consistent with the hypothesis that, at least within our local epidemiology, MBL interspecies transfer among these species may primarily occur via transposable elements, rather than through direct plasmid transfer. Nevertheless, the differences observed with other studies further support the theory that transmission of antimicrobial resistance gene determinants between these species may occur in diverse ways, depending on the mobile genetic elements circulating in each epidemiological context.

SNP analysis revealed close genetic relatedness among ST69 *P. alloputida*, ST114 *P. kurunegalensis* and ST115 *P. mosselii* isolates, respectively. Nevertheless, only two spatiotemporal overlaps between patients were identified in *P. kurunegalensis*, effectively ruling out direct patient-to-patient transmission among the patients in whom *P. alloputida* and *P. mosselii* isolates were identified. Except for the two detected epidemiological links between *P. kurunegalensis* isolates (P07–P19 and P10–P16, Figure 1C), epidemiological analysis did not reveal evidence of sustained patient-to-patient transmission in the other MBL-NAP and MBL-STZ isolates, suggesting that most cases likely represent independent acquisition events. This finding highlights the effectiveness of ongoing microbiological surveillance and infection control measures in preventing clonal spread within the hospital setting. However, one pALO03-carrying *P. mosselii* isolate recovered from an ICU sink drain differed by 30 whole-genome SNPs from a pALO03-carrying *P. mosselii* isolate obtained during the same month from a tracheal aspirate of a patient hospitalized in the ICU, suggesting a potential acquisition event originating from an environmental source. Similarly, a study performed in a hospital in The Netherlands described a *P. putida* outbreak which included nine patients hospitalized in a hematology-oncology unit, and the source was an environmental reservoir linked to the hospital's drainage system (van der Zwet et al., 2022). These findings highlight the important role of indirect nosocomial transmission routes such as environmental reservoirs in the hospital environment, and underscore the importance of systematic environmental surveillance in hospital settings, particularly in units where antimicrobial use is extensive and the proportion of immunocompromised patients is substantial.

### 4.1 Limitations

This study presents some limitations that should be acknowledged. First, its single-center design may not allow the findings to be generalized to other healthcare settings with different epidemiological backgrounds. Third, AST results were interpreted using clinical breakpoints defined for *P. aeruginosa* or other non-Enterobacterales due to the absence of clinical breakpoints for the species identified in this study, which may have influenced MIC interpretation. Fourth, resistance mechanisms to aztreonam and aztreonam/avibactam could not be determined, as the functional validation of predicted resistance mechanisms was beyond the scope of this study. Therefore, the proposed contribution of efflux-mediated or other non-enzymatic mechanisms in aztreonam/avibactam resistance remains inferential. Finally, only one of the *bla*_VIM_-harboring plasmids identified could be assigned to a described incompatibility group, reflecting both the extensive plasmid diversity among non-*aeruginosa Pseudomonas* and *Stutzerimonas* species and the current lack of well-characterized plasmid classification systems for these organisms. In addition, the number of MBL-producing *Stutzerimonas* isolates and environmental *P. mosselii* isolates was limited, reflecting their low incidence in our setting, as all available isolates during the study period were included. Despite this, the genomic approach applied provides relevant insights into the genetic context and potential interspecies dissemination of antimicrobial resistance determinants.

## 5 Conclusions

In summary, our results indicate that, in addition to ongoing efforts to prevent the spread of carbapenemase-producing *P. aeruginosa* in healthcare settings, it is very important to prioritize both clinical and environmental surveillance of other carbapenemase-producing *Pseudomonas* species within the hospital environment. Despite their comparatively lower clinical significance, these species may still be involved in nosocomial transmission events and represent an important reservoir of transmissible antimicrobial resistance determinants. In addition, more studies are required to evaluate the interspecies transmission of antimicrobial resistance genes from non-*aeruginosa Pseudomonas* and *Stutzerimonas* species to *P. aeruginosa* and vice versa, in order to better understand the host range of the mobile genetic elements circulating among these species in different epidemiological contexts.

## Data Availability

The datasets presented in this study can be found in online repositories. The names of the repository/repositories and accession number(s) can be found in the article/Supplementary material.

## Supplementary material

Supplementary Figure 1 Structural organization of pSOL01 plasmid.

Supplementary Figure 2 Structural organization of pSTU02 plasmid.

Supplementary Figure 3 Structural organization of pALO03 plasmid.

Supplementary Figure 4 Structural organization of pSTU06 plasmid.

Supplementary Figure 5 Structural organization of pOLE015 plasmid.

Supplementary Figure 6 Structural organization of pASI017 plasmid.

Supplementary Figure 7 Structural organization of pASI021 plasmid.

Supplementary Table 1 Metadata of the patients and studied metallo-β-lactamase-producing non-*aeruginosa Pseudomonas* and *Stutzerimonas* genomes obtained at our hospital from March 2022 to July 2024.

Supplementary Table 2 Metadata of the studied VIM-producing P. aeruginosa genomes obtained at our hospital between 2022 and 2024.
