# Supplementary figures and images for: Emerging reservoirs of metallo-β-lactamase genes among non-aeruginosa Pseudomonas and Stutzerimonas species in a Spanish tertiary hospital
Source: Front Microbiol. 2026 May 22;17:1810036. doi: 10.3389/fmicb.2026.1810036 (PMC13261756; doi:10.3389/fmicb.2026.1810036)

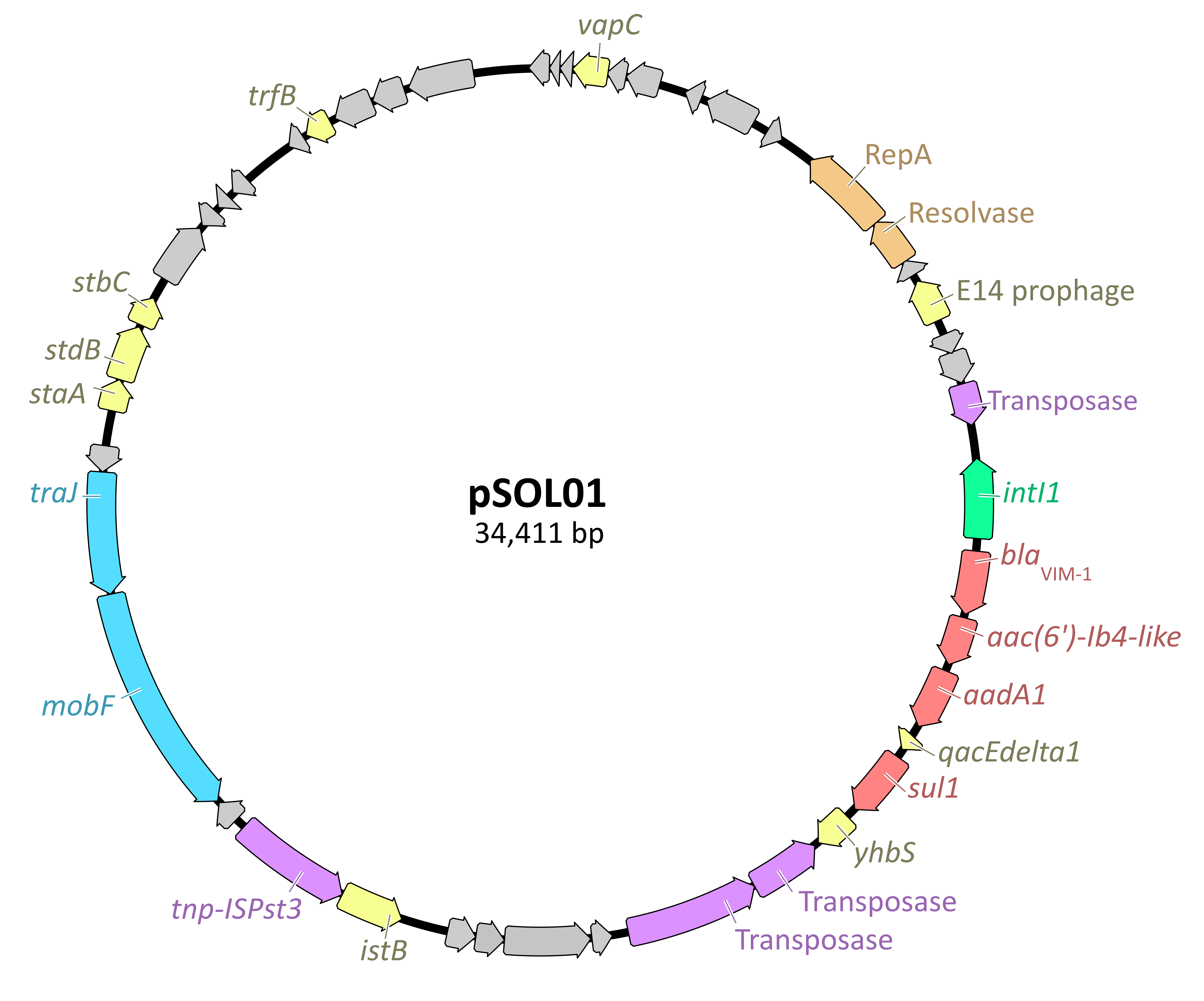

Supplement: Supplementary Figure 1 — Structural organization of pSOL01 plasmid. [file Image_1.tiff]

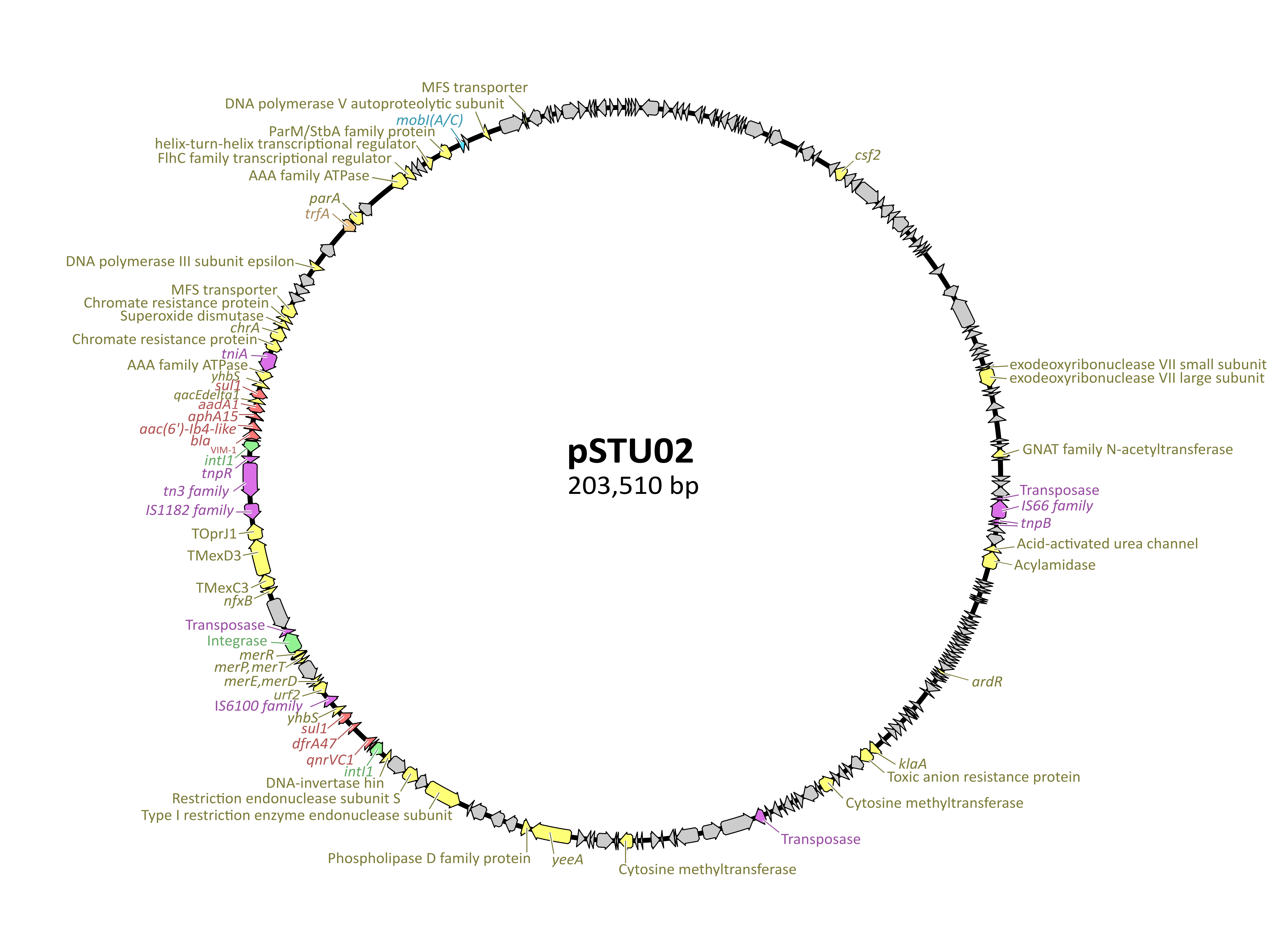

Supplement: Supplementary Figure 2 — Structural organization of pSTU02 plasmid. [file Image_2.tiff]

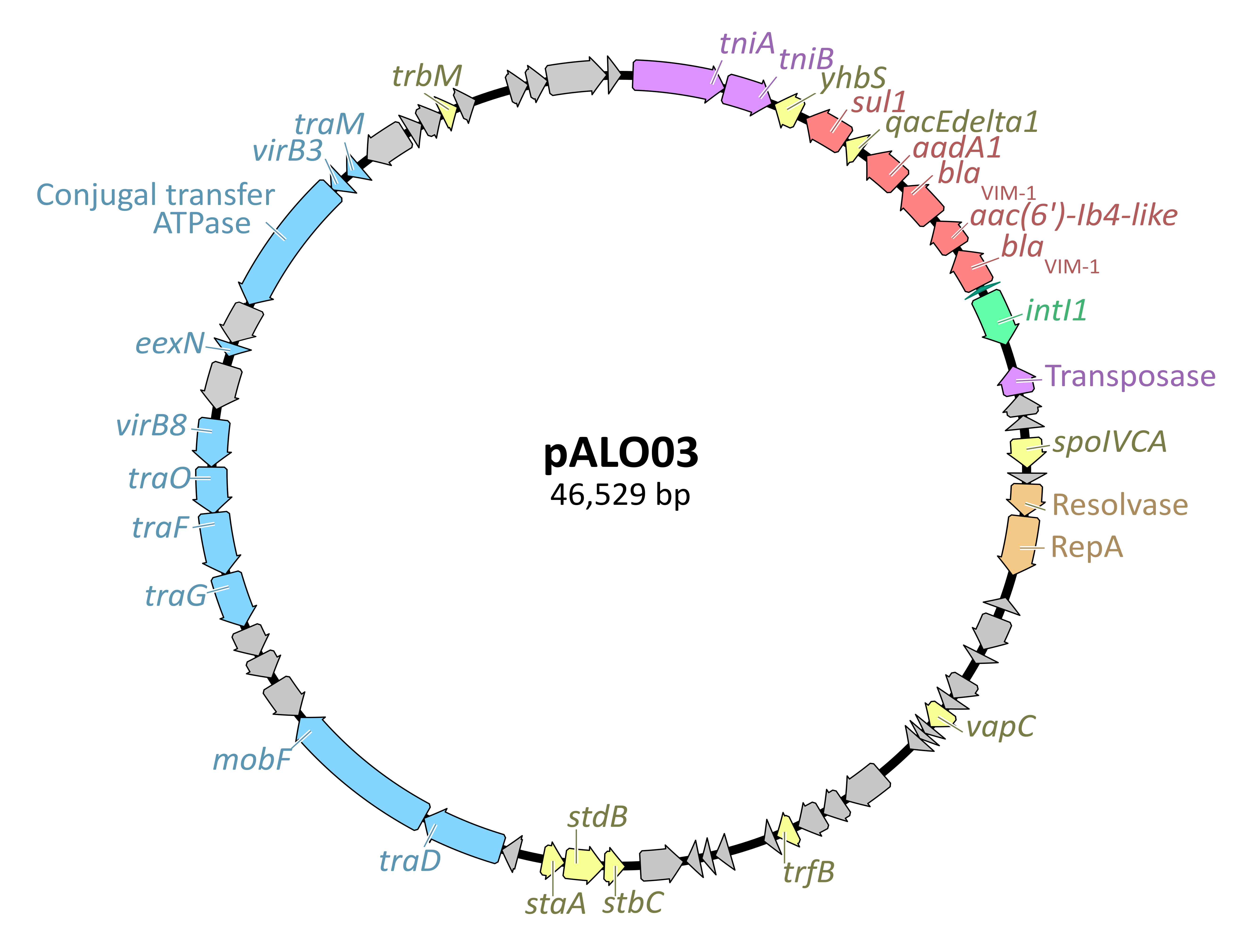

Supplement: Supplementary Figure 3 — Structural organization of pALO03 plasmid. [file Image_3.tiff]

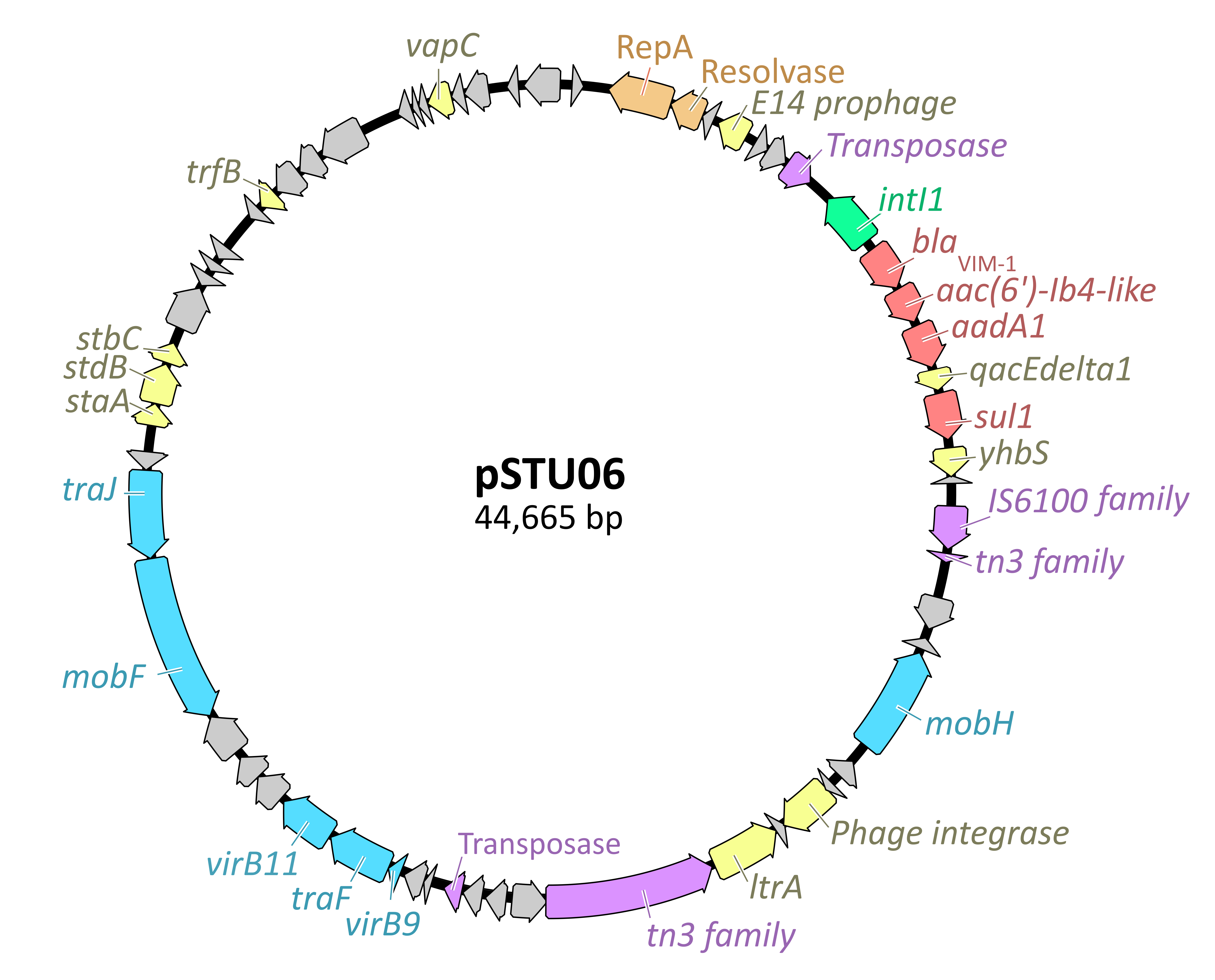

Supplement: Supplementary Figure 4 — Structural organization of pSTU06 plasmid. [file Image_4.tiff]

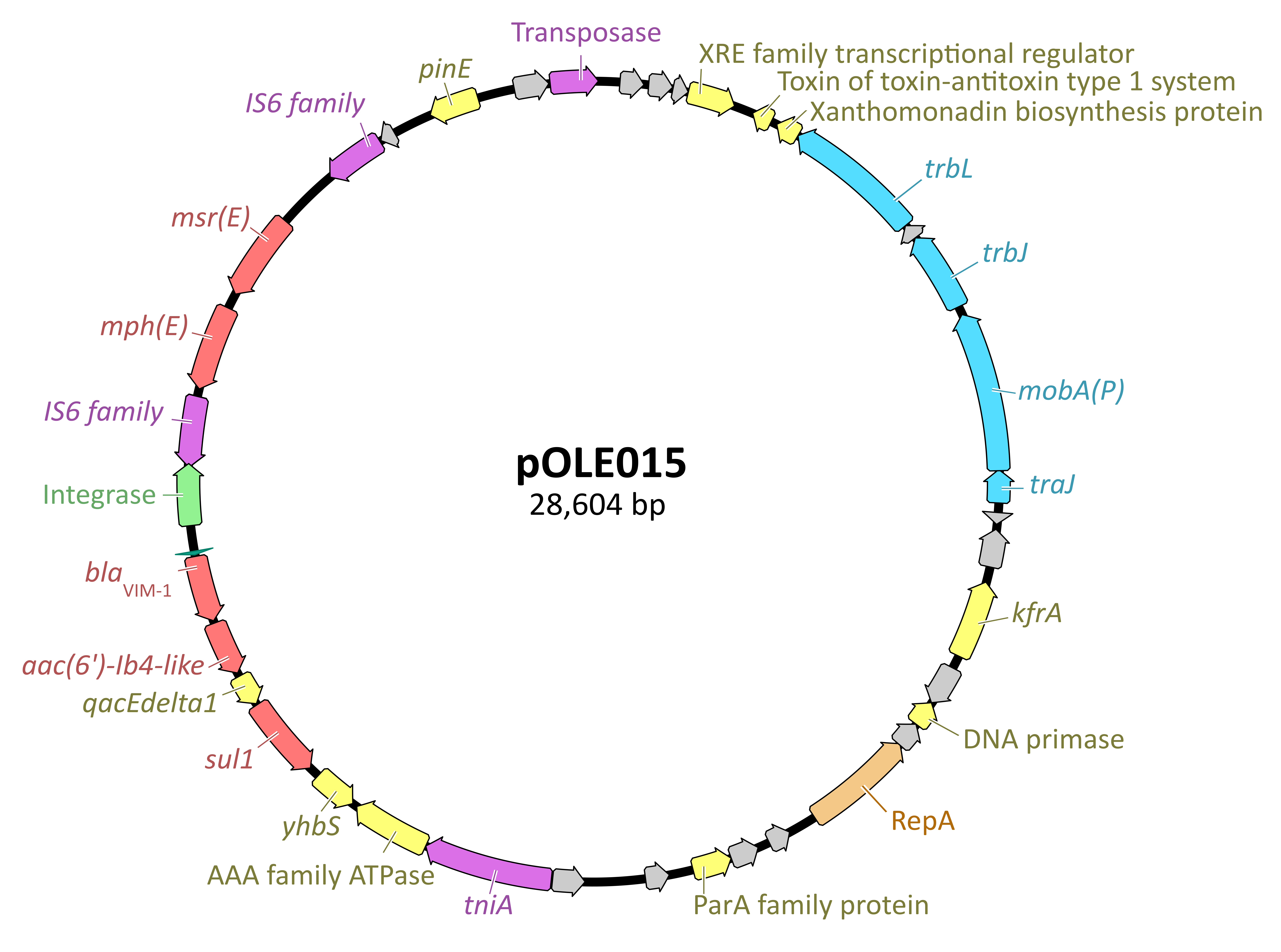

Supplement: Supplementary Figure 5 — Structural organization of pOLE015 plasmid. [file Image_5.tiff]

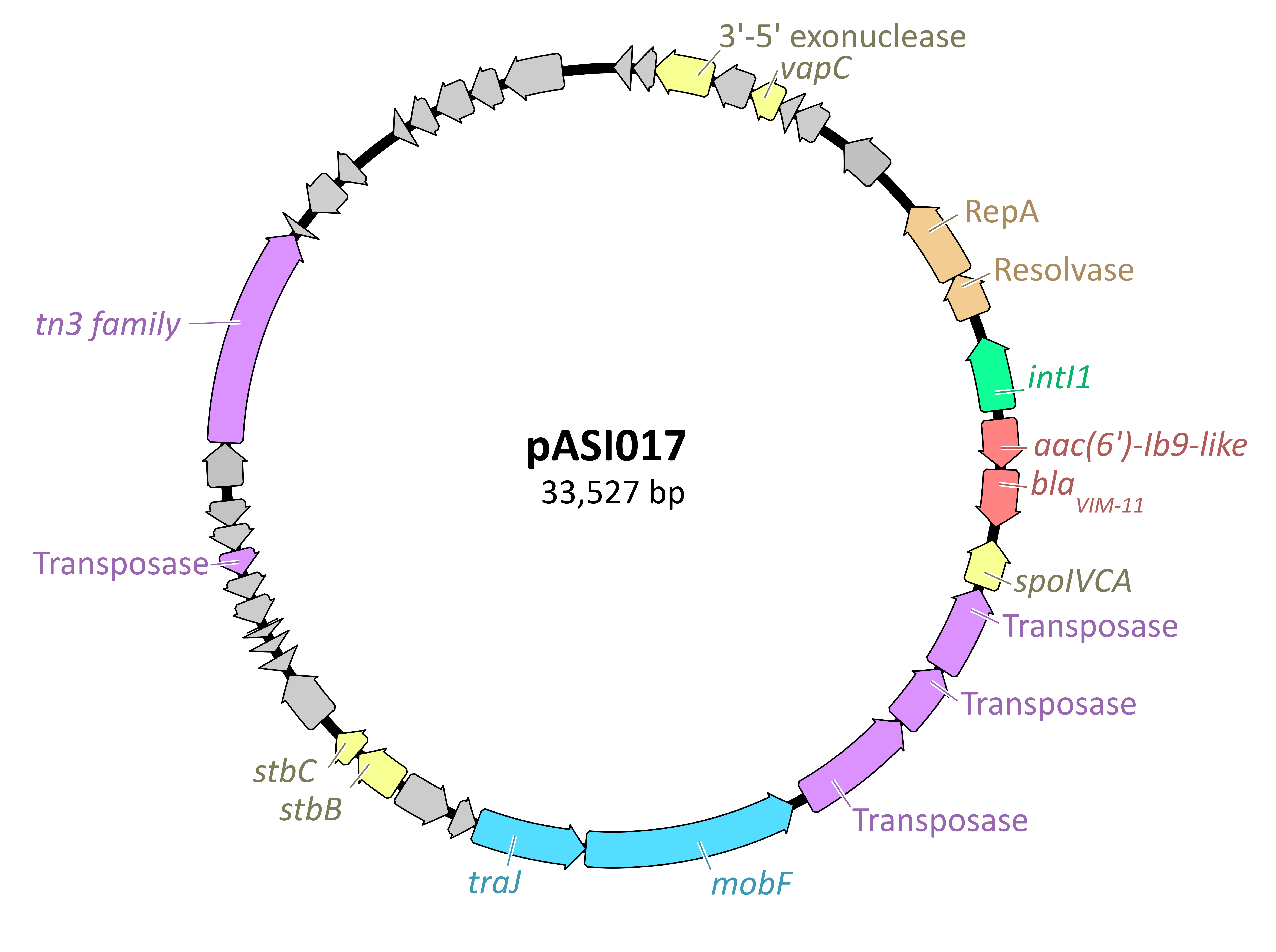

Supplement: Supplementary Figure 6 — Structural organization of pASI017 plasmid. [file Image_6.tiff]

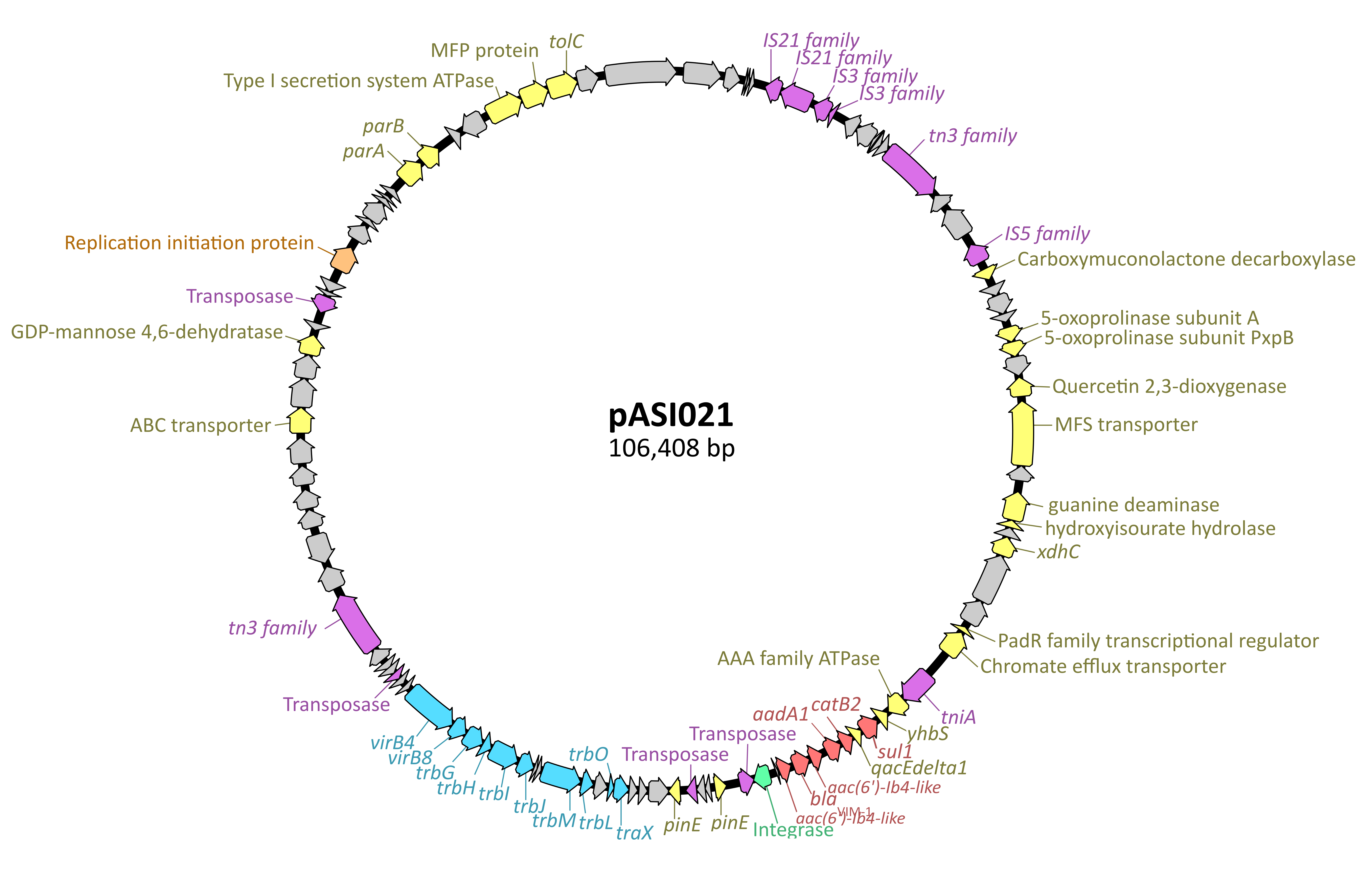

Supplement: Supplementary Figure 7 — Structural organization of pASI021 plasmid. [file Image_7.tiff]
